# Outer retina changes on optical coherence tomography in vitamin A deficiency

**DOI:** 10.1186/s40942-020-00224-1

**Published:** 2020-06-05

**Authors:** Meghan K. Berkenstock, Charles J. Castoro, Andrew R. Carey

**Affiliations:** Johns Hopkins School of Medicine, Wilmer Eye Institute, 600 N. Wolfe Street, Woods 459A, Baltimore, MD 21287 USA

**Keywords:** Vitamin A deficiency, Optical coherence tomography, Nyctalopia

## Abstract

**Background:**

Vitamin A deficiency is rare in the United States and can be missed in patients with malabsorption syndromes without a high dose of suspicion. Ocular complications of hypovitaminosis A include xerosis and nyctalopia, and to a lesser extent reduction in visual acuity and color vision. Outer retinal changes, as seen on spectral domain optic coherence tomography (SD-OCT), in patients with vitamin A deficiency have previously not been documented.

**Case presentation:**

We present two cases with symptoms of severe nyctalopia who were subsequently diagnosed with severe Vitamin A deficiency and their unique findings on SD-OCT of outer nuclear layer diffuse thinning with irregular appearance of the interdigitating zone and the ellipsoid zone as well as normalization after vitamin A supplementation.

**Conclusions:**

Outer nuclear layer thinning and disruption of the outer retinal bands on SD-OCT are reversible with correction of vitamin A deficiency. Improvement in visual acuity, color vision, and nyctalopia are possible with early diagnosis and appropriate treatment.

## Background

Most commonly seen in regions with food insecurity, nutritional deficiencies, or restricted diets, vitamin A deficiency is rare in developed countries [1–9]. However, the increase in bariatric surgeries in response to the obesity epidemic in the United States has created a new population susceptible to vitamin A deficiency through malabsorption [10–18]. For the same reason, patients with chronic pancreatitis and inflammatory bowel disease are at risk for the development of vitamin A deficiency [19, 20. Without a high index of suspicion in both of these groups, the deficiency can remain undetected.

Vitamin A deficiency can lead to a well-known array of ocular complications, including nyctalopia, xerosis with Bitot spots, and xanthopsia [9, 21–23]. If detected early, oral or intramuscular vitamin A replacement can reverse ocular complications prior to permanent vision loss [16, 24, 25]. Only a few reports have described the photoreceptor changes on spectral domain optical coherence tomography (SD-OCT) [26–28]. Here we describe a cohort of patients with malabsorption syndromes with outer retinal abnormalities on SD-OCT in the setting of vitamin A deficiency.

## Case presentations

### Case 1

A 71-year-old white male presented with a 3-year history of nyctalopia, most notable when driving through tunnels and needing a flashlight to see at night. His past medical history was significant for a remote history of colon cancer status post a partial bowel resection. Recently, he was diagnosed with an inoperable gastrointestinal carcinoid for which he was on somatostatin due to omental metastases. The ocular history was notable for dry eyes of 3 years. On examination, his visual acuities without correction were 20/30 in the right eye (OD) and 20/40 in the left eye (OS). Color vision was reduced on Ishihara plate testing to 1.5 out of 10 in OD and 2 out of 10 in OS. Noted on slit lamp examination was the presence of iris neovascularization without involvement of the angle in OD. Fundus examination was negative for neovascularization of the disc or retinal hemorrhages. SD-OCT of the macula demonstrated diffuse thinning of the outer retina with an irregular appearance of the interdigitating zone and inner-outer segment junction (ellipsoid zone) in both eyes (OU) most significantly in the temporal parafovea. Serum vitamin A level was not detectable at < 5 mcg/dL. The lower limit of normal = 38 mcg/dL.

**Fig. 1:**
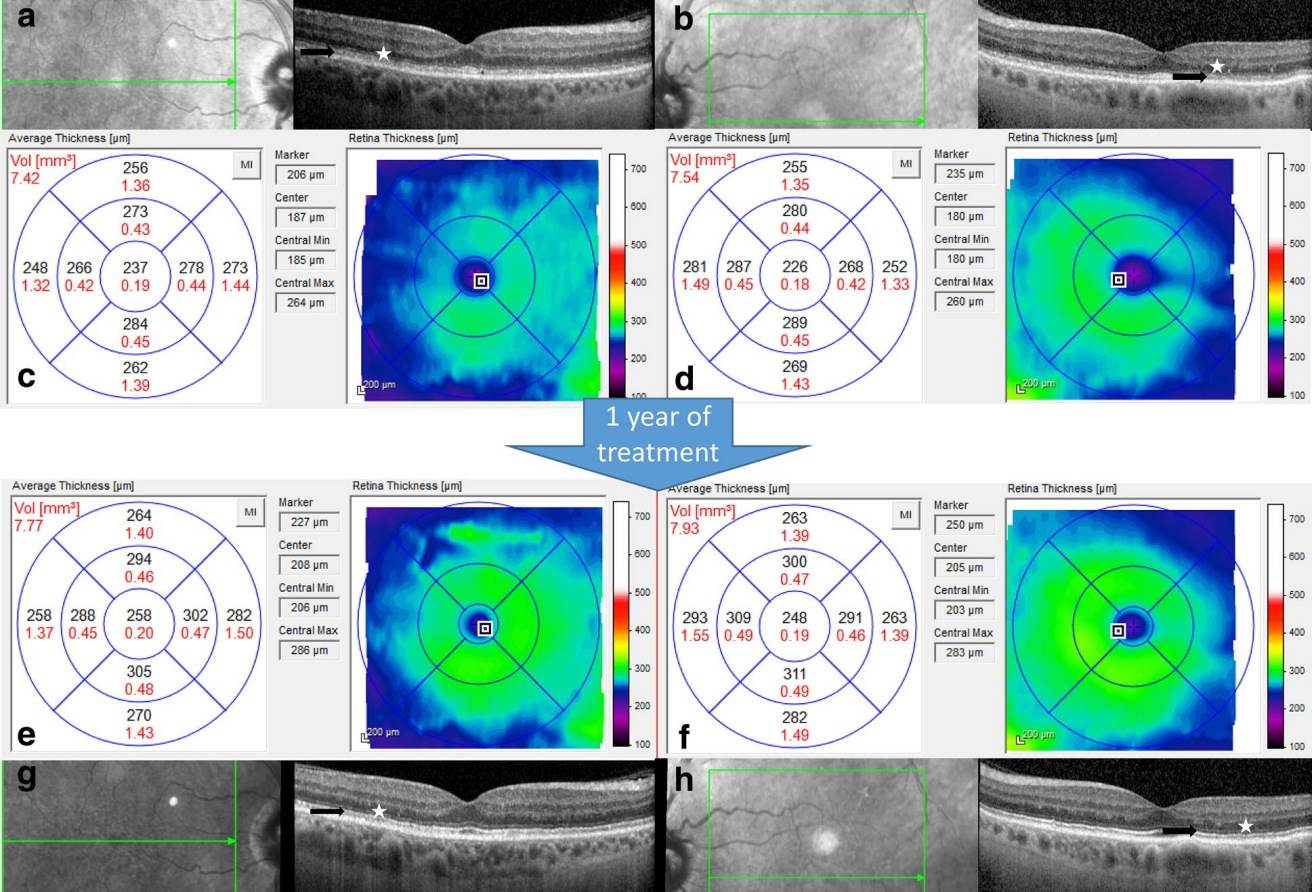
Macula SD-OCT changes from baseline through 1 year of treatment. **a**, **b** SD-OCT Horizontal line scan through fovea at baseline of right & left eye respectively (black arrow highlights ratty appearance of ellipsoid zone and white star highlights thin outer nuclear layer). **c**, **d** SD-OCT Volume scans at baseline of right and left eye respectively. **e, f** SD-OCT Horizontal line scan through fovea at 1-year follow-up of right and left eye respectively (black arrow highlights reconstituted appearance of ellipsoid zone and white star highlights improved outer nuclear layer). **g**, **h** SD-OCT Volume scans at one-year follow-up of right and left eye respectively. *SD-OCT* spectral domain optical coherence tomography

He was started on oral vitamin A supplementation with 56,000 international units (IU) daily with noticeable improvement in his visual acuity and night vision within several days. Follow-up examination one month after starting treatment showed an improved visual acuity of 20/20 OU. SD-OCT demonstrated mild retina thickening mostly of the outer nuclear layer but also appreciated on total retinal volume measurements as well as reconstitution of the outer retinal bands. Repeat serum Vitamin A level had improved to 30 mcg/dL. After three months, the improvements on SD-OCT had plateaued with increase of 25–50% in the parafoveal outer nuclear layer thickness, but his color vision improved to 5/10 OU after 6 months. Given difficulty with adherence to oral vitamin A replacement and serum Vitamin A levels remaining between 26 and 35 mcg/dL, he was transitioned to monthly intramuscular (IM) injections of 100,000 international units. Afterwards, his ocular exam remained stable. The OCT changes at baseline and follow-up can be seen in Fig. 1.

### Case 2

**Fig. 2:**
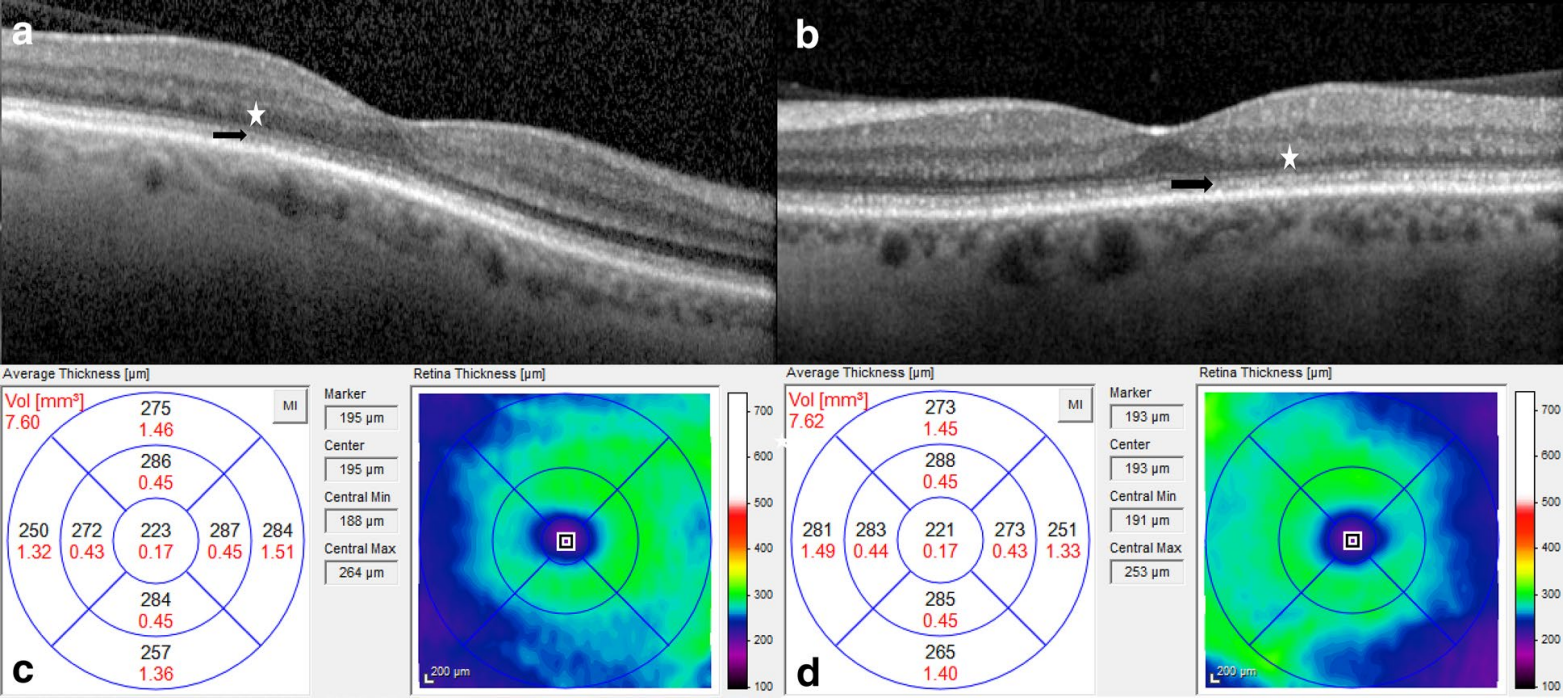
Macula SD-OCT changes at baseline. **a**, **b** SD-OCT Horizontal line scan through fovea at baseline of right & left eye respectively (black arrow highlights ratty appearance of ellipsoid zone and white star highlights thin outer nuclear layer). **c**, **d** SD-OCT Volume scans at baseline of right and left eye respectively. *SD-OCT* spectral domain optical coherence tomography

A 57-year-old white male who presented with 2-year history of slowly progressive dimming of his vision. He noted the need for more light and high contrast text to read. He also noted a dimming of his peripheral vision, difficulty with color vision, and nyctalopia for 1 year. His past medical history was significant for acute on chronic pancreatitis attributed to a combination of alcohol abuse and gallstones with pancreatic insufficiency, secondary diabetes mellitus, chronic obstructive pulmonary disease, chronic kidney disease, HIV, and a two-year history of hydroxychloroquine use with cumulative dose 292 g which was discontinued 1 month prior to presentation. On examination, he had uncorrected visual acuities of 20/40 OU and on Ishihara color plate testing, he identified 1 out of 13 plates OU. At the slit lamp, bilateral punctate corneal epithelial erosions and attenuated retinal arterioles were noted. SD-OCT of the macula demonstrated diffuse thinning of the outer nuclear layer with an irregular appearance of the interdigitating zone and inner-outer segment junction (ellipsoid zone) in both eyes (Fig. 2). Goldmann perimetry was near normal in both eyes with the size III4e isopter showing a horizontal diameter of 110° in each eye. Serum vitamin A level was 13 mcg/dL with a lower limit of normal of 33 mcg/dL. The patient was started on oral vitamin A replacement of 100,000 IU and referred back to his primary care provider and local retinal specialist for monitoring as he lived 292 miles and 2 states away. On phone follow-up he reported improvement in subjective vision acuity, color vision, night vision, and peripheral vision after the start of replacement therapy.

## Discussion and conclusions

The extramacular manifestations of vitamin A deficiency, including Bitot spots and xerosis, are widely known and can be identified readily on slit lamp examination. With the advent of newer retinal imaging techniques, we have a more in-depth view of retinal anatomy in the setting of chronic disease. Aleman et al. and Nishida et al. simultaneously were the first to report structural changes on SD-OCT in vitamin A deficiency in December 2013 [26, 28]. Saenz-de-Viteri et al. demonstrated the macular thinning and improvement with treatment in 2016, but this is the first paper to localize these changes to the outer nuclear layer [27]. With extended follow-up and normalization of vitamin A levels, outer nuclear layer and overall macular thickness increased on repeat scans.

While vitamin A is typically attributed to rod function, the foveal thinning along with decreased color vision and reduced visual acuity indicate cone dysfunction in addition. Focal disruption of the photoreceptor outer segments with a hyperreflective signal on SD-OCT has been reported [26]. The areas of hyperreflectivity were located near yellow flecks near the arcades in the posterior pole on dilated fundus exam. Retinal flecks have been previously reported in hypovitaminosis A [29, 30]. The neighboring ellipsoid zone and retinal pigment epithelium bands remained intact and no changes to the inner retina were noted. The authors hypothesized that the spots seen in the posterior pole correspond to both loss and accrual of photoreceptor segments secondary to less phagocytosis by RPE cells in vitamin A deficiency [26]. A second case of outer retinal changes on SD-OCT was reported in a patient with hereditary vitamin A deficiency due to a bi-allelic mutation in *RBP*-*4* [31]. While there were no retinal flecks, the loss of the ellipsoid and inner and outer segment junction was again noted. In our first case, we show that these changes are reversible with repeat imaging over an extended follow-up and vitamin A repletion. Furthermore, we suggest that the irregular appearance of the interdigitating zone and inner-outer segment junction reflect rod and cone dysfunction prior to the accumulation of photoreceptor segments, which lead to the development of white spots on exam and hyperreflective images on SD-OCT.

Compared with the previous literature on vitamin A deficiency, both cases in this series require discussion due to the concomitant use of somatostatin and hydroxychloroquine. In addition to bowel resection, the patient in case 1 was also on somatostatin, which has been shown to cause malabsorption of the fat soluble vitamins [32]. This may have contributed to the chronic need for such high dose vitamin A replacement via parenteral methods. However, continued treatment with somatostatin was necessary to prevent progression of his cancer. The patient in case 2 had vision symptoms beginning around the same time as treatment with hydroxychloroquine was initiated. Toxicity prior to 5 years of use of hydroxychloroquine or a cumulative dose of 1000 grams is less than 0.3%, and the patient only had a cumulative dose of 292 g [33]. Therefore, we feel the hydroxychloroquine was unlikely to play a contributory role to the visual symptoms of the patient or SD-OCT changes.

This study is limited by small numbers and limited follow-up. Additionally, eletro-retinography were not used to correlate response to anatomic or functional responses to treatment. Further research could involve a larger number of patients, pre-operative evaluation undergoing malabsorptive bariatric surgery, use of electro-retinography, and longer follow-ups.

Although rare in the United States, the number of patients at risk for vitamin A deficiency is growing. Early diagnosis can reverse structural changes at the level the photoreceptors, leading to improved visual acuity and color vision. SD-OCT offers a view of the cellular underpinnings leading to vision loss in vitamin A deficiency which may help to confirm diagnosis and monitor response to treatment.

## Data Availability

Not applicable

## Abbreviations

IM: Intramuscular
IU: International units
OD: Right eye
OS: Left eye
OU: Both eyes
SD-OCT: Spectral domain optical coherence tomography
